# Supplementary material for: In situ atomic-scale observation of grain size and twin thickness effect limit in twin-structural nanocrystalline platinum
Source: Nat Commun. 2020 Mar 3;11:1167. doi: 10.1038/s41467-020-14876-y (PMC7054541; doi:10.1038/s41467-020-14876-y)
Supplement: Supplementary file 1 — Supplementary Information [file 41467_2020_14876_MOESM1_ESM.pdf]

In situ Atomic-scale Observation of Grain Size and Twin Thickness Effect Limit in Twin-structural Nanocrystalline Platinum

Wang et al

Supplementary Figures

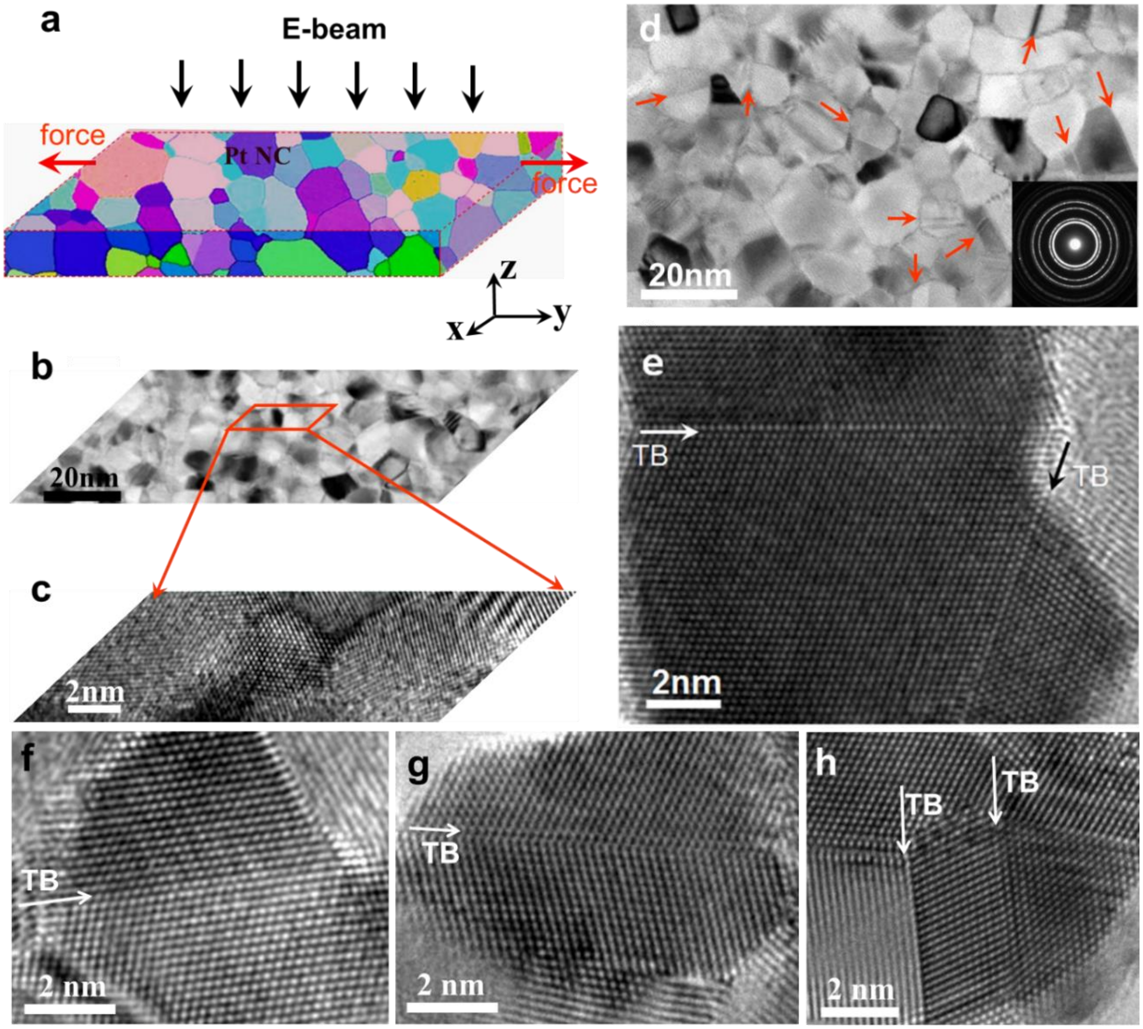

Supplementary Figure 1. TEM observations of the twin-structural nanocrystalline Pt thin film. (a) Schematic view illustrating the deformation method with our newly developed double-tilt TEM tensile stage. (b, c) With this special TEM tensile stage, the NC Pt thin film can slowly and gently deform and simultaneously retain the double-tilt capability. Thus, the grains can be oriented appropriately, and the atomic-scaled deformation process can be recorded during loading (c). (d) The bright-field TEM image shows that the film consists of nano-sized and equiaxed grains, and many grains contain growth twins. The selected area diffraction pattern in

the inset indicates the films without obvious preferred orientation. (e-h) Four typical HRTEM images show that the TBs are perfect, with no pre-existing dislocation.

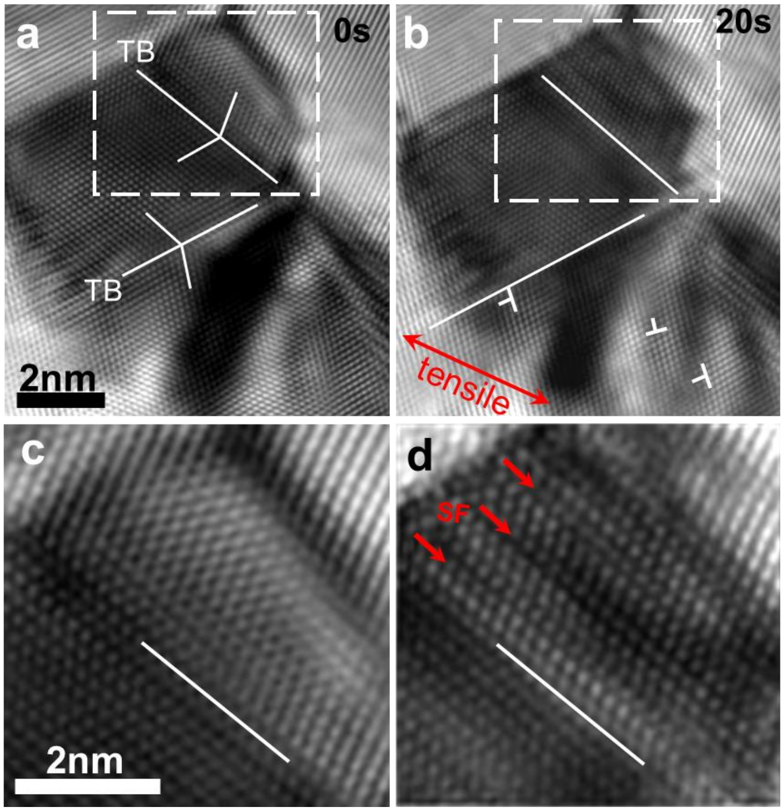

Supplementary Figure 2. *In situ* observation of partial dislocation nucleated in thin twins and full dislocation nucleated in thick twins in a grain. (a, b) Three full dislocations marked with T nucleated in the thick twin. (c, d) Enlarged HRTEM image taken from the framed region of (a) and (b), respectively. The partial dislocation emission resulted in stacking faults in the 3 nm thickness twin.

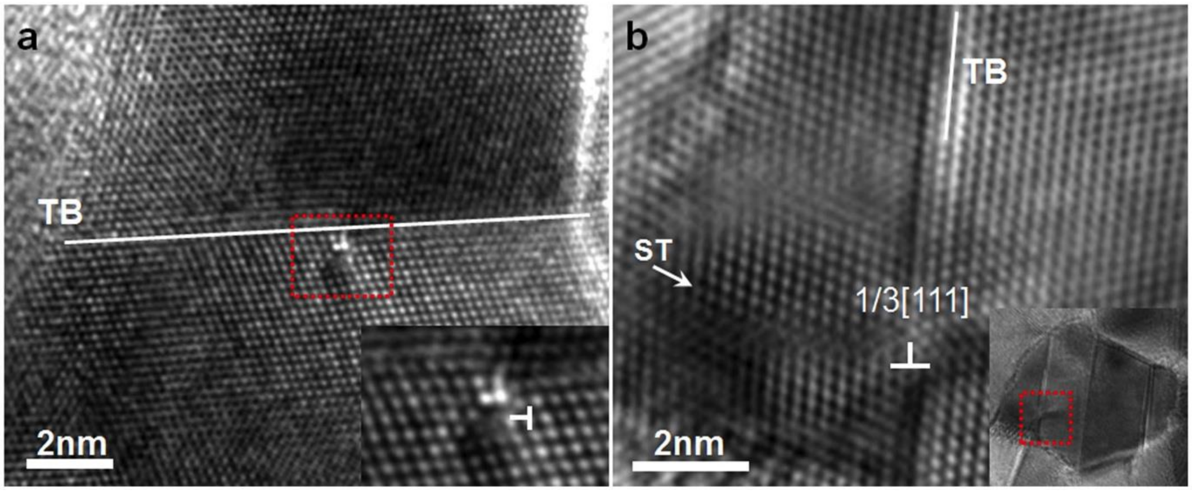

Supplementary Figure 3. Two examples show the full dislocation nucleation in thick twins in large grains. (a) HRTEM image showing a full dislocation obstacle by a TB. Inset: Enlarged HRTEM image clearly showing the dislocation core. (b) Enlarged HRTEM image corresponding to the red framed region of the inset, showing a full dislocation obstacle by the TB. Inset: low magnified TEM image showing the twin-structured nanograin.

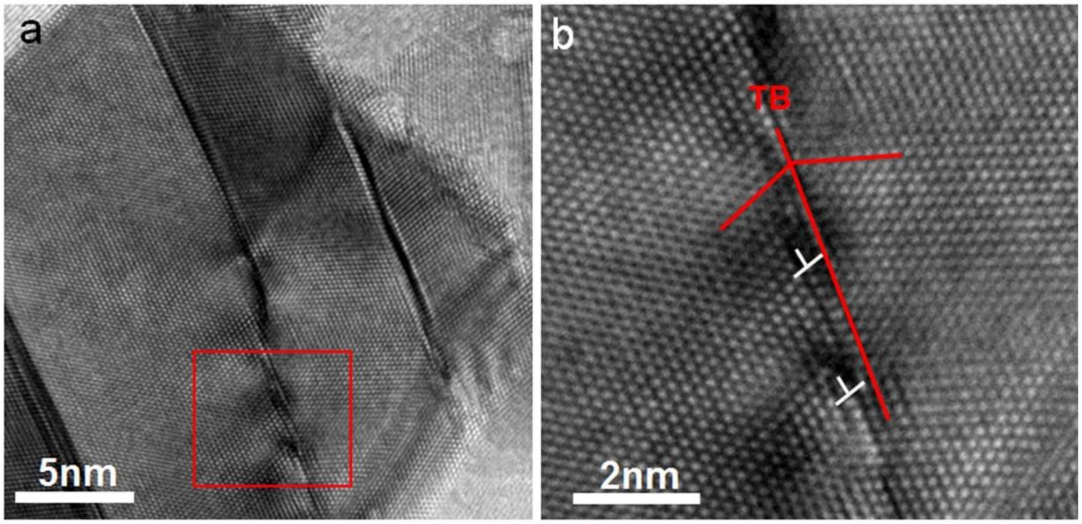

Supplementary Figure 4. Another example shows full dislocation in thick twin lamellae for a grain with *d* ~21 nm. (a) HRTEM image showing a full dislocation obstacle by TB. (b) Enlarged HRTEM image corresponding to the red framed region of (a) shows the atomic structure of full dislocations obstacle by TB, as marked with T.

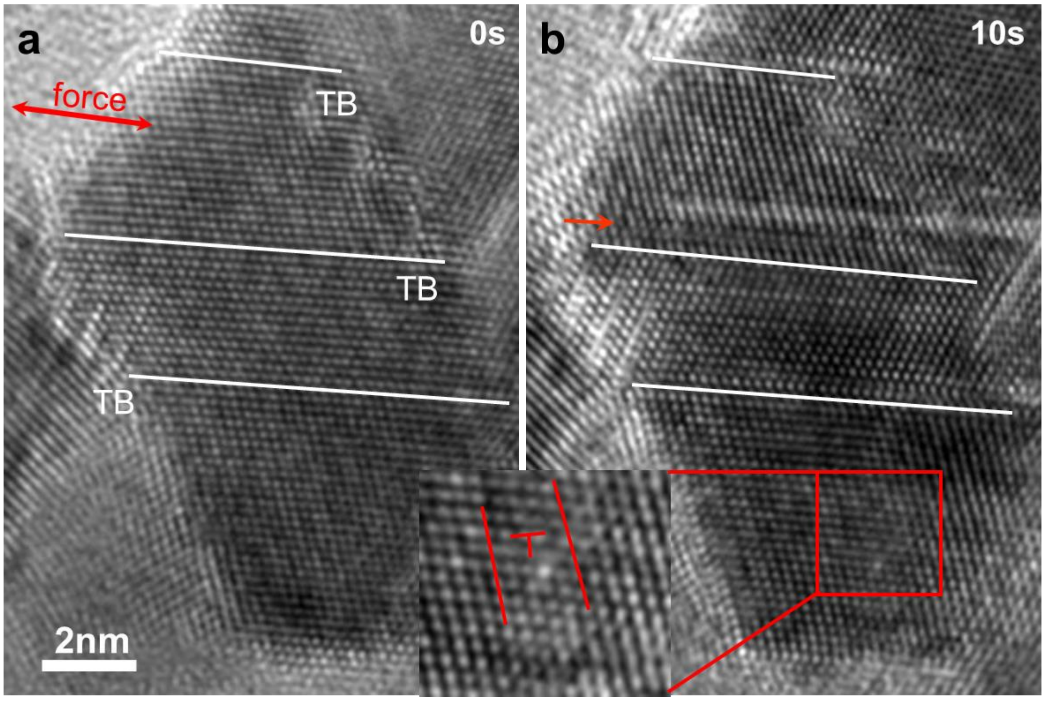

Supplementary Figure 5. *In situ* observation of both partial and full dislocation was in a grain containing both thin and thick twin lamellae. (a, b) Two HRTEM images captured 10 s apart. Full dislocation in thick twins (as marked with T) and SF resulting from partial dislocation emission from GBs (as noted by arrow) was observed.

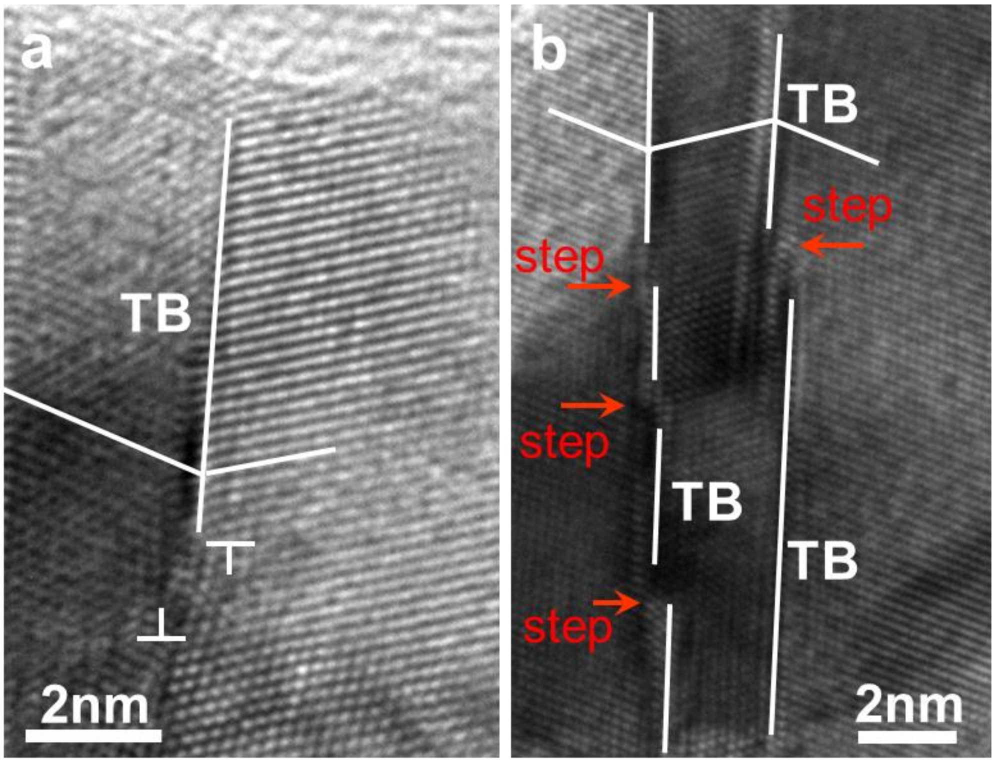

Supplementary Figure 6. Two other examples show the switch from partial dislocation to full dislocation in the grains. (a) HRTEM image showing the full dislocation obstacle by TBs in a thick twin, as marked with T. (b) In a thin twin, SF and stepped TBs that resulted from the partial dislocations were observed.

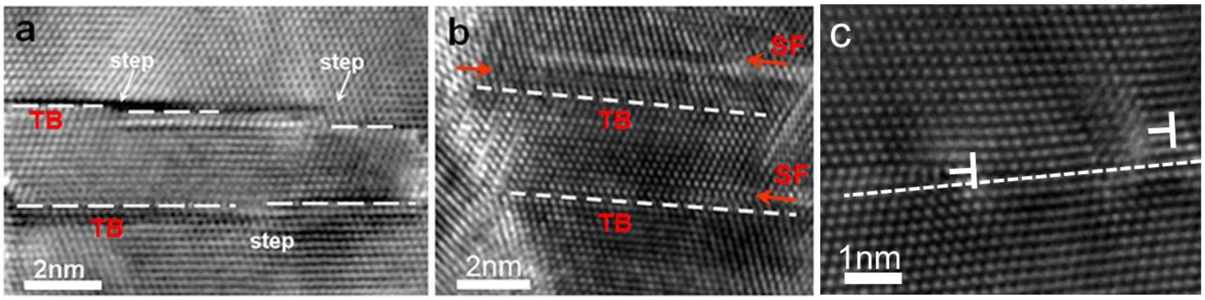

Supplementary Figure 7. Three examples show the switch from partial dislocation to full dislocation. (a, b) In a thin twin, SF and stepped TBs that resulted from the partial dislocations were observed. (c) Two full dislocations obstacle by TBs in a thick twin, as marked with T.

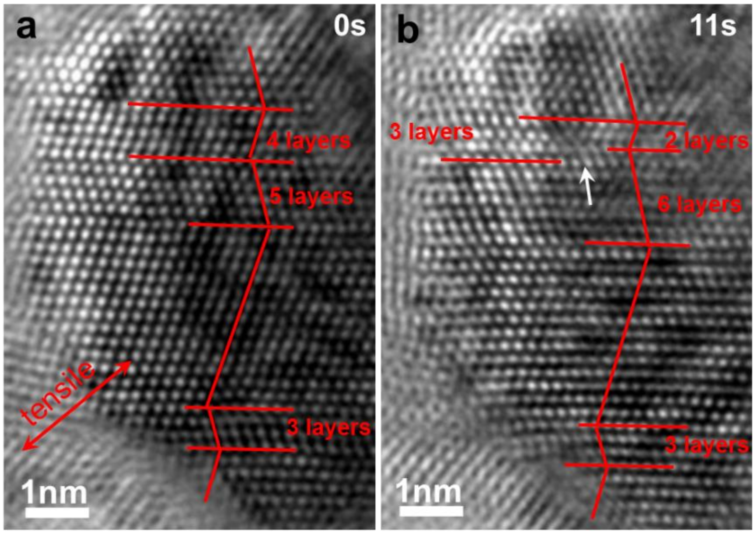

Supplementary Figure S8. Typical *in situ* HRTEM images show TB migration caused by partial dislocation nucleation and glide on the plane parallel to TBs in an ~ 7 nm grain. (a) HRTEM image taken when the straining was initially loaded on the twin-structured grain; the 4 atomic-layer and 5 atomic-layer thick twins are atomically flat. (b) With further straining, the twin thickness changed, and a step on the TB caused by partial dislocation was observed. For these small-sized grains, only partial dislocations parallel to the TB were observed.

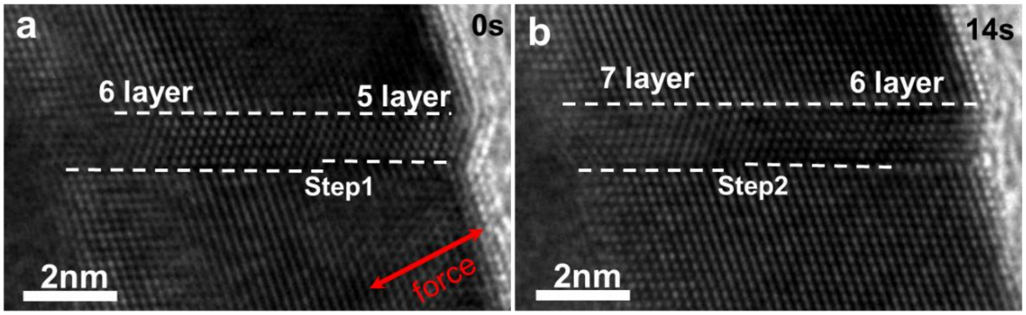

Supplementary Figure 9. *In situ* observation of partial dislocation nucleation and glide on the plane parallel with TBs in an ~ 8 nm sized grain.

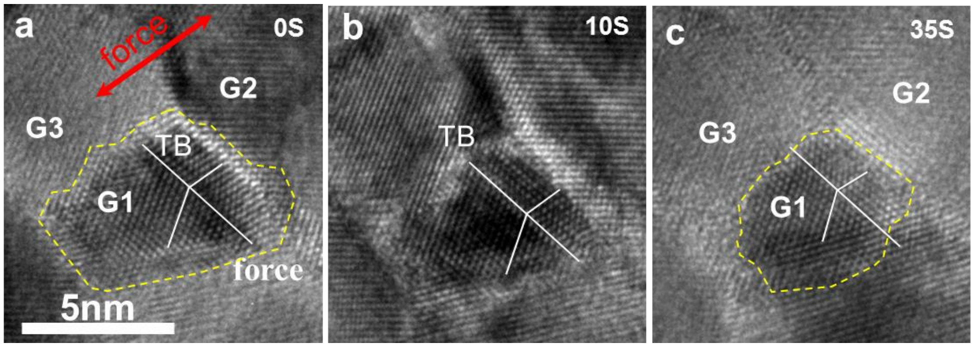

Supplementary Figure 10. Direct observation of GB-mediated plasticity when twin-structural grains are smaller than ~ 6 nm. (a–c) HRTEM images captured during tensile loading, showing a ~4 ×6 nm-sized grain shrinking into an ~3 ×4.5 nm-sized grain resulting from GB migration. As can be observed, G_2 and G_3 exhibit fringe change, indicating that G_2 and G_3 underwent slightly out-of-plane rotation.

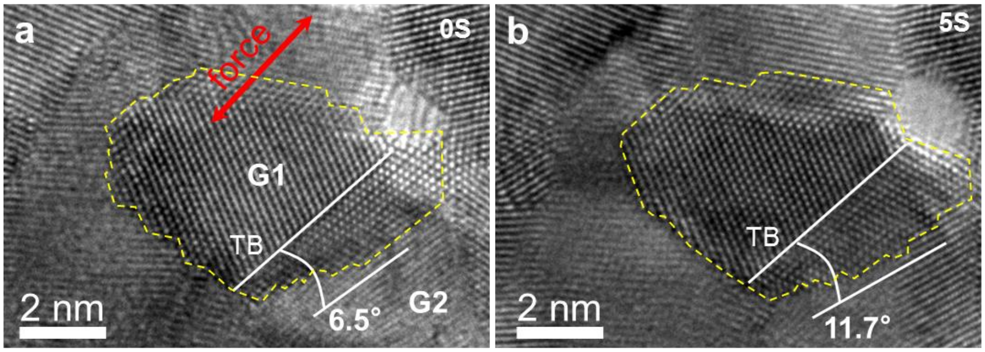

Supplementary Figure 11. *In situ* observation of GB migration in small-sized twin-structural grain. The angle between the lattice/fringe in G1 and G2 increased from 6.5 ° to 11.7 °, indicating that there is in-plane rotation during the GB migration in this case.

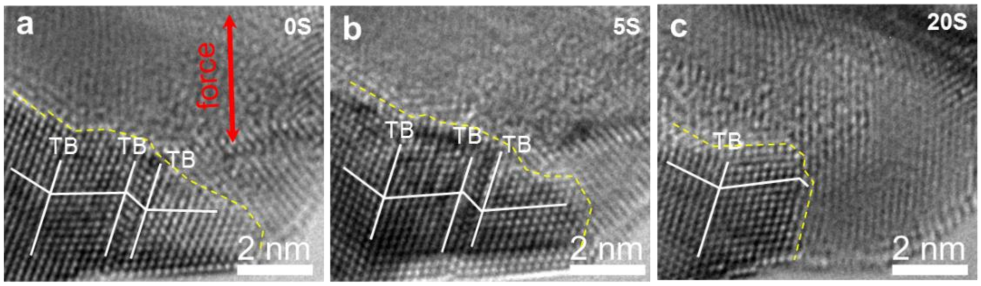

Supplementary Figure 12. *In situ* observation of GB migration in small-sized twin-structural grain.

Supplementary Note 1

The critical TT for a given grain size. According to a previous theoretical prediction [1], the dependence of flow stress on both TT and grain size for twin-structured metals is the following:

$$\tau = \frac{KT}{SV}\ln[\frac{\lambda \dot{e}}{dv_D}\exp(\frac{VU}{KT})] \qquad (1)$$

Where τ is the flow shear stress, K is the Boltzmann constant, T *is* the temperature, S is a factor representing local stress concentration in the range of ~ 1.2, V is dislocation activation volume, ν_D is the Debye frequency in the range of $1.0\times10^{13}s^{-1}$, ΔU *is* the activation energy, λ is TT, $\dot{e}$ is strain rate, and d is grain size.

The Hall-Petch dependence of flow stress on grain size is as follows:

$$\tau = \tau_0 + \frac{k}{\sqrt{d}} \qquad (2)$$

According to previous experimental studies, the τ_0 is about 0.001G, where G is Young's modulus of materials. $k = 3266\text{MPa}\sqrt{\text{nm}}$ and this is nearly the same for the FCC metals [2].

According to equation (1) and (2), the critical TT for a given grain size is as follows:

$$\frac{k}{\sqrt{\text{d}}} + \frac{KT}{SV}\ln(\frac{dv_D}{\lambda \dot{e}}) = \frac{VU}{SV} - \tau_0 \qquad (3)$$

In our experiment, T *is* ~ 330K and $\dot{e}$ is ~ 10^{-3}. According to previous theory [3], V is ~ $3b^3$ and ΔU is 1.2eV. According to equation (3), the calculated critical TT is ~ 6.7 nm for the grain with a size of 10 nm, while it is ~ 8.5nm for the grain with a size of 20nm.

Supplementary Note 2

The transition from full to partial dislocation. The transition from full to partial dislocation in small-sized grains and thin twins/matrixes can be understood by comparing the shear stress required to nucleate a perfect dislocation to the one required to initiate partial dislocation. According to a previous prediction, the dislocation nucleation stress can be written as follows [4, 5]:

$$\tau_N = 2\mu b_N/d \quad (4)$$

and

$$\tau_P = 2\mu b_P/d + \gamma/b_P \quad (5)$$

Where τ_N and τ_P are the critical shear stresses needed to nucleate a full and partial dislocation, d is the grain size, μ is the shear modulus, γ is the stacking fault energy, and b_N and b_P are the magnitudes of the Burgers vectors of the full and partial dislocation, respectively. According to equations (1) and (2), there is a critical grain size d_c, below which the partial dislocation needs a lower stress nucleation than that in full dislocations:

$$d_c = 2\mu(b_N - b_P)b_P/\gamma \quad (6)$$

For Pt, taking the SF energy as 0.27 to 0.373 Jm^{-2} [6, 7] and the shear modulus as 65.2 GPa [8], the calculated critical size d_c is ~ 7–9.5 nm. For Cu, the calculated critical d is about 33–88 nm when taking SF energy of 0.02 to 0.053 Jm^{-2} [6, 7] and shear modulus of 54.8 GPa [8] into account. For Ag, the calculated critical d is ~ 122.8 nm when taking SF energy of ~ 0.018 Jm^{-2} [6, 7] and shear modulus of 56.7 GPa [8] into account.

Supplementary References:

1. Li, X. Y., Wei, Y. J., Lu, L., Lu K. & Gao, H. J. Dislocation nucleation governed softening and maximum strength in nano-twinned metals. *Nature* **464**, 877-880 (2010).

2. Zhu, T., Li, J., Samanta, A., Leach, A. & Gall, K. Temperature and strain-rate dependence of surface dislocation nucleation. *Phys. Rev. Lett.* **100**, 025502 (2008)

3. Greer, J. R. & De Hosson, J. T. M. Plasticity in small-sized metallic systems: Intrinsic versus extrinsic size effect. *Prog. Mater. Sci.* **56**, 654-724 (2011).

4. Chen, M. W., Ma, E., Hemker, K. J., Sheng, H. W., Wang, Y. M. & Cheng, X. M. Deformation twinning in nanocrystalline aluminum. *Science* **300**, 1275-1277 (2003).

5. Zhu, Y. T., Liao, X. Z. & Wu, X. L. Deformation twinning in nanocrystalline materials. *Prog. Mater. Sci.* 57, 1-62 (2012).

6. N. Bernstein and E. B. Tadmor, *Phys. Rev. B* **69**, 094116 (2004).

7. M. J. Mehl, D. A. Papaconstantopoulos, N. Kioussis and M. Herbranson, *Phys. Rev. B.* **61**, 4894 (2000)

8. D. H. Warner, W. A. Curtin and S. Qu, *Nat. Mater.* **6**, 876 (2007).
